# Prevalence and Risk Factors of Anemia During Pregnancy in Saudi Arabia: A Systematic Review

**DOI:** 10.7759/cureus.49287

**Published:** 2023-11-23

**Authors:** Ahmed Mustafa, Ghaida Alanazi, Maisa Alanazi, Ahlam Alenezi, Manal Alenzi, Fay Al-Muteri, Abeer H Aljohani, Ghazir A Alruwili, Rehab Almatrafi, Shuruq Mohsen A Alshammari

**Affiliations:** 1 Obstetrics and Gynecology, Maternity and Children Hospital, Arar, SAU; 2 Medicine, Northern Border University, Arar, SAU; 3 Family and Community Medicine, Northern Border University, Arar, SAU; 4 Family Medicine, Maternity and Children Hospital, Arar, SAU; 5 College of Medicine, Northern Border University, Arar, SAU; 6 General Medicine, Northern Border University, Arar, SAU; 7 General Practice, Northern Border University, Arar, SAU

**Keywords:** ksa, systematic review, saudi arabia, pregnancy, anemia

## Abstract

The prevalence of anemia during pregnancy in Saudi Arabia is variable. Nulliparous, multiparous >3, and multi-gravidity are associated risk factors with a higher incidence of anemia during pregnancy. Other risk factors comprised working, women in university, past history of anemia, obesity, women younger than 25 years, low income, longer menstrual cycle >5 days, bleeding during pregnancy, reduced birth spacing, a low level of education, and decreased intake of iron-rich foods.

## Introduction and background

Anemia during pregnancy is a prevalent concern, characterized by a decrease in the blood's capacity to transport oxygen, primarily due to a reduction in hemoglobin levels. This decline can be either absolute or relative in nature. It is widely acknowledged that the majority of pregnancies lead to a greater increase in plasma volume compared to red blood cell mass, resulting in what is termed “physiologic anemia.” This phenomenon, historically referred to as “plethora gravidarum,” has been recognized for centuries to describe these physiological changes. The debate surrounding whether this “hydremia” is within the bounds of normalcy or represents a pathological condition remains ongoing [1-3].

The prevalence of anemia among pregnant women in Saudi Arabia ranges from 18% to 58% among pregnant women in different parts of the country. These rates may also vary based on factors such as socioeconomic status, access to healthcare, and dietary habits. Overall, anemia in pregnancy remains a significant public health concern in Saudi Arabia [2].

A hematocrit of less than 33% or a hemoglobin level of less than 11 g/dL at any point during pregnancy is considered anemia of pregnancy, according to the World Health Organization (WHO) [1]. The US Centers for Disease Control and Prevention classifies anemia of pregnancy as having hemoglobin levels less than 11 g/dL, hematocrit levels less than 33% in the first or third trimester or having hemoglobin levels less than 10.5 g/dL, or hematocrit levels less than 32% in the second trimester [3].

With each stage of pregnancy, the risk of anemia increases. According to CDC standards, among American low-income pregnant women, in the first trimester, 8% of women are anemic; in the second, 12%; and the third, 34% [4]. According to the US Department of Health and Human Services (DHHS), the prevalence of third-trimester anemia is a significant indicator of reproductive health. With regard to prevalence, African Americans have the highest prevalence rate (48.5%), subsequently followed by Whites (27.5%), Asians, Native Hawaiians, and other Pacific Islanders (29%), Hispanics and Latinas (30.1%), American Indians and Alaska Natives (33.9%), and Hispanics and Latinas [4,5].

Doctors have known for a long time that hydremia alone cannot explain why 10% to 70% of pregnant women described in early 20th-century research had hemoglobin levels less than 7 g/dL. The 1950s demonstrated a significant function for iron deficiency in pregnancy anemia due to the frequent detection of hypochromia, microcytosis, and anisocytosis in blood smears of pregnant women with anemia and the correction of such anomalies following the administration of iron supplements [6]. Since then, the iron shortage has been acknowledged as the most prevalent cause of anemia in pregnancy around the world. This anemia typically manifests in the third trimester, when the iron is most maximally collected to support erythropoiesis in the developing baby [7].

This systematic review aims to study the prevalence and associated risk factors with anemia in pregnancy among Saudi women. Our results are anticipated to provide specific guidance for future studies and what needs to be addressed to fill in knowledge gaps at this time.

## Review

Methodology

Investigating the prevalence and risk factors for anemia in pregnancy among Saudi women is the objective of this systematic review. To locate the pertinent literature, a thorough search was conducted across four significant databases, including PubMed, Web of Science, EBSCO, and Cochrane Library, while restricting our search to English and taking into account each database's specific requirements. The following keywords were converted into PubMed Mesh terms and used to find the relevant studies: “Anemia,” “Iron deficiency anemia,” “Pregnancy,” “Pregnant women,” “Maternal,” “Saudi Arabia,” and “KSA.” To match the necessary keywords, the Boolean operators “OR” and “AND” were applied. The search returned a list of publications containing complete English text, free papers, and human trials.

Inclusion criteria

Studies that determined the prevalence of iron deficiency anemia in pregnancy and risk factors, studies that included Saudi women, and studies in the English language.

Exclusion criteria

Women with underlying chronic disease and women with chronic types of anemia, e.g., sickle cell anemia.

We applied Rayyan (QCRI) to detect duplicates in the output of the search technique [8]. To assess the appropriateness of the titles and abstracts, the researchers narrowed the combined search results based on a set of inclusion/exclusion criteria. The papers that matched the requirements for inclusion were carefully read by the reviewers. The writers discussed methods for settling disagreements. A data extraction form was constructed, and it was used to upload the approved study. The study titles, authors, city, participant count, mean age, prevalence of anemia, potential risk variables, and primary outcomes were all extracted by the authors. Utilizing the information gathered from the pertinent studies, summary tables were made to provide a qualitative analysis of the outcomes and study components covered. The most effective method for utilizing the data from the included study articles was selected after data for the systematic review had been extracted.

Using the ROBINS-I risk of bias assessment method for non-randomized trials of treatments, the included studies' quality was assessed [9]. The seven themes that were assessed were confounding, participant selection for the study, intervention classification, intervention deviations from intended interventions, missing data, outcome evaluation, and choice of the reported result.

Results

**Figure 1:**
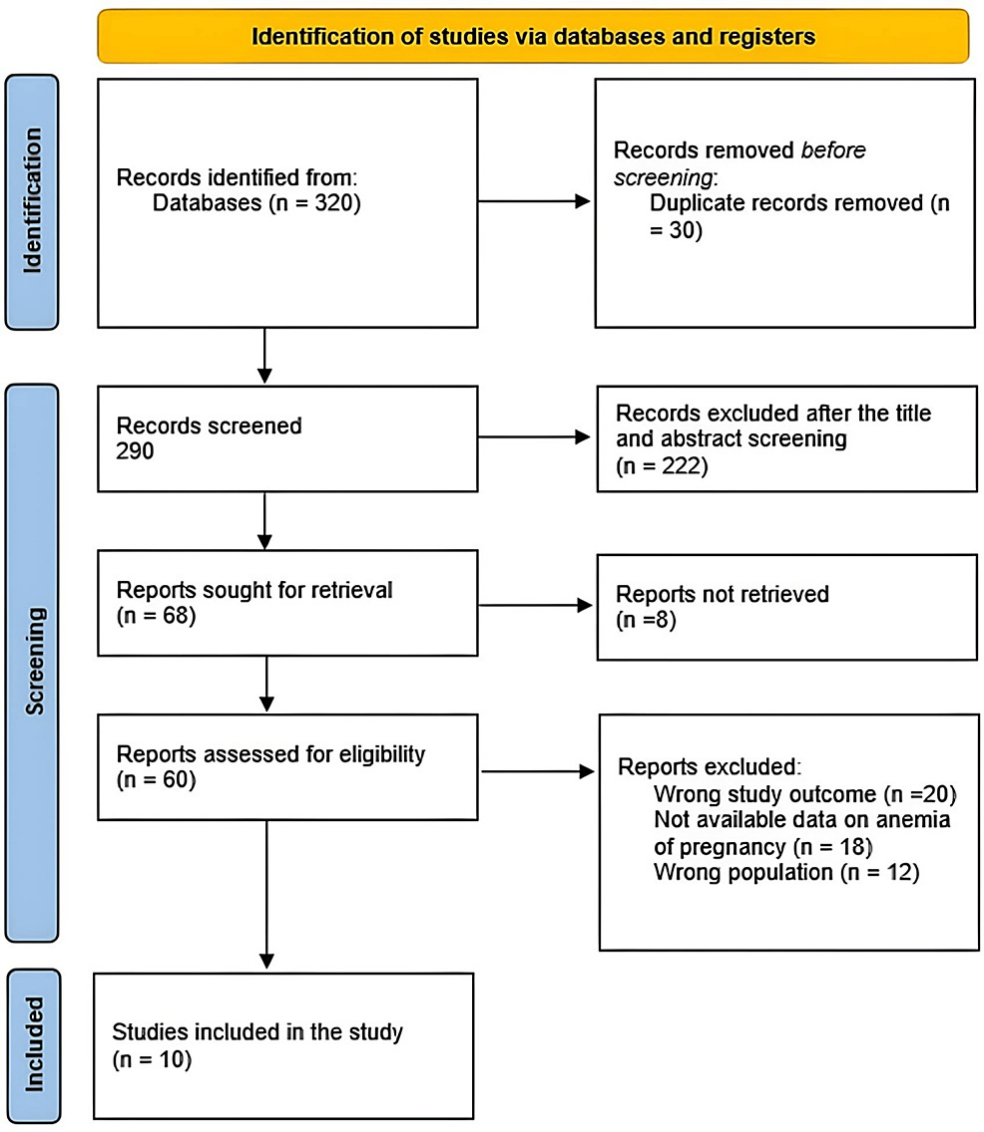
PRISMA flowchart summarizes the study selection process.

The systematic search produced 320 study articles in total after removing 30 duplicates. Two thousand ninety studies were subjected to title and abstract screening; 222 studies were disregarded. Only 8 of the 68 reports that were searched for recovery could not be found. The final analysis of 60 papers revealed that 20 had erroneous research outcomes, 18 lacked information on anemia of pregnancy among Saudi women, and 12 had an inappropriate population type. This systematic review contained 10 appropriate study papers. Figure 1 displays a summary of the study selection procedure.

Characteristics of the included studies

Table 1 includes the sociodemographic characteristics. A total of 10 studies with 9,950 patients were included [10-19]. Two studies were conducted in Riyadh [10,14], two in Jeddah [11,17], one in Arar [12], one in Hail [13], one in Jazan [15], one in Makkah [16], one in Al-Khobar [18], and one in Taif [19]. The prevalence of anemia in pregnancy in Saudi Arabia ranged from 16% [19] to 82.9% [12]. The reported risk factors to increase the incidence of anemia in pregnancy included nulliparous [10,12], while multigravida/multiparous was a risk factor in four studies [13-15,19]. Other risk factors included working, women in university [12,13], history of anemia [11,13,16], obesity [17], women younger than 25 years [10], low income [13], longer menstrual cycle > 5 days [13], bleeding during pregnancy [13], infrequent intake of meat [13], reduced birth spacing [16], a low level of education [16], and decreased intake of iron-rich foods [18].

The included studies reported that anemia remains a major issue for pregnant women [10-19]. Antenatal or prenatal screening for anemia should be individualized for each pregnant person [11,19]. Two studies reported the importance of compliance for iron supplementation combined with adequate intake of iron-rich dietary sources during pregnancy and for three months after delivery should be covered in health education programs at the PHCCs [12,18], as illustrated in Table 1.

**Table 1:** Sociodemographic and clinical characteristics and outcomes of the included studies.

Study	Study design	City	Participants	Mean age	Prevalence (%)	Risk factors	Key findings	ROBIN-I
Mahfouz et al., 1994 [10]	Cross-sectional	Riyadh	372	29.3±5.8	20.4	Nulliparous, third trimester, women < 25 years, working and university women had a higher prevalence of anemia	Anemia continues to be a major issue for pregnant women, endangering their prognoses	Moderate
Fallatah et al., 2020 [11]	Retrospective	Jeddah	5120	29.9±6	55.6	Abnormal hemoglobin levels, past anemia, blood transfusion, intrauterine fetal death, and stillbirth	Antenatal or prenatal screening for anemia should be individualized for each pregnant person based on her health and overall clinical judgement	Moderate
Alanazi et al., 2019 [12]	Cross-sectional	Arar	299	NM	82.9	More than half of working women had anemia during pregnancy. Primigravida women are slightly more likely to develop anemia than multiparous women	The data emphasized the value of iron supplements for expectant mothers, highlighting the need for community awareness programs	High
Alreshidi et al., 2021 [13]	Cross-sectional	Hail	390	29.9±7.6	34.1	Low income, bigger family size, higher parity, longer menstrual cycle > 5 days, bleeding during pregnancy, infrequent intake of meat, the habit of drinking tea just after meals, and past history of anemia	These results help healthcare professionals understand the value of early detection and treatment of anemia in early pregnancy.	Moderate
Baradwan et al., 2018 [14]	prospective cohort	Riyadh	1579	29	44.5	Gravidity >8 and parity of >3	Compliance with iron supplementation was seen to prevent negative outcomes for the mother and fetus	Moderate
Salih et al., 2015 [15]	Cross-sectional	Jazan	389	15-44 (range)	58.9	Multigravida women	Many desires and aversions that affect nutrition occur in pregnant women	Moderate
Abdelhafez et al., 2012 [16]	Cross-sectional	Makkah	100	15-55 (range)	39	Reduced birth spacing, a low level of education, and a past history of anemia	As part of a comprehensive health promotion strategy, health providers must devote more attention to teaching pregnant women healthy long-term eating habits	Moderate
Fallatah et al., 2019 [17]	Retrospective	Jeddah	1037	31.96±5.8	59.4	Obesity	The mother and fetus suffered negative effects as a result of maternal obesity. Hence, periconceptional counselling, performing health education, and implementing a complete plan prior to pregnancy should be mandated	Moderate
Rasheed et al., 2008 [18]	Cross-sectional	Al-Khobar	464	26.7±5.4	41.4	Decreased intake of iron-rich foods on average	The importance of compliance for iron supplementation combined with adequate intake of iron-rich dietary sources during pregnancy and for three months after delivery should be covered in health education programs at the PHCCs.	Moderate
Hafez et al., 2014 [19]	Cross-sectional	Taif	200	33.6±4.7	16	Previous pregnancies and obesity	The study advocated using appropriate screening methods and ongoing medical education and in-service training for every member of the primary health care team for recognizing high-risk pregnancies	Moderate

Discussion

Anemia is related to maternal physical and psychological comorbidity, as well as a higher risk of neonatal and maternal morbidity and mortality [20]. The current study results indicate a wide variation in the prevalence of anemia among Saudi pregnant women. The records show that the Eastern region of Saudi Arabia has the highest prevalence of anemia in pregnant women. The wide variation in the prevalence could be due to cultural diversity in socioeconomic conditions, lifestyle, and health-seeking practices, anemia in pregnancy is more common than one may think. A recent systematic review and meta-analysis reported that the worldwide prevalence of anemia during pregnancy of 36.8% (95% CI: 31.5%-42.4%), and the highest prevalence was in Africa (41.7%) [21,22].

This review reported that the risk factors to increase the incidence of anemia in pregnancy included nulliparous [10,12], while multigravida/ multiparous >3 was a risk factor in four studies [13-15,19]. This might result from multiple pregnancies depleting a pregnant woman's iron reserves [23]. Grand multigravida women had a greater percentage of anemia (66.7%), according to a Malaysian study [24].

Multiparity is a significant risk factor linked to iron deficiency anemia, claim Okafor et al. and Isah et al. [25,26]. The iron shortage will develop from having too many pregnancies too soon apart because pregnancy uses up a lot of iron [27]. The need for iron during pregnancy is three to four times greater than that of non-pregnant women [28]. Women, in general, are observed to have low iron stores, perhaps as a result of the monthly blood loss during menstruation, even though it can be mobilized from the maternal stores to meet this requirement [29]. The mother would get iron deficiency after these reserves are gone [30]. Iron deficiency anemia can develop from a decrease in the hemoglobin production rate caused by an iron deficiency [31]. Women's iron deficiency anemia can be managed by reducing the overall number of pregnancies and lengthening the period between pregnancies. Family planning and child spacing will lower a woman's need for iron, preventing iron depletion and the effects of iron deficiency anemia.

Other risk factors comprised working, women in university [12,13], past history of anemia [11,13,16], obesity [17], women younger than 25 years [10], low income [13], longer menstrual cycle >5 days [13], bleeding during pregnancy [13], infrequent intake of meat [13], reduced birth spacing [16], a low level of education [16], and decreased intake of iron-rich foods [18]. The occurrence of iron deficiency anemia in pregnancy has also been linked to low socioeconomic variables [32]. Since anemia is inversely correlated with the socioeconomic level of households, particularly in developing contexts, it serves as a marker of socioeconomic deprivation [33].

According to the included research, anemia is still a significant problem for pregnant women [10-19]. Each pregnant woman should get personalized antenatal or prenatal anemia screening [11,19]. According to two studies, health education programs at PHCCs should cover the need for compliance for iron supplementation and adequate intake of iron-rich dietary sources during pregnancy and for three months following delivery [12,18]. Iron and folic acid supplementation should be a priority for the government and non-governmental organizations as part of standard antenatal care for all pregnant women. In Saudi Arabia's regions with a higher prevalence of anemia, it is crucial to employ long-acting family planning techniques to prevent frequent pregnancies. Health extension workers should promote antenatal follow-ups and community-based awareness campaigns. Further investigations conducted around the country are required to comprehend the causes of anemia in pregnant women.

Strengths and limitations

The following are some strengths of this thorough literature review: In order to avoid reviewer biases as much as possible, we feel that (a) we followed PRISMA recommendations and used reputable methods for data extraction and quality evaluation, and (b) we presented all study findings graphically to the reader. We also mention the following restrictions: In our search, we cannot completely rule out the potential of missing some studies. But we also looked through a number of databases, reference books by hand, and grey literature. The heterogeneity of the studies prevented us from performing a meta-analysis. The ability of analyses to attain significance may be constrained by the small sample sizes and methodological problems in some of the included research. The validity and generalizability of the results in some studies with small sample numbers and high rates of loss-to-follow-up may be compromised by selection bias.

## Conclusions

The prevalence of anemia during pregnancy among Saudi women was variable. Multiparous >3, multi-gravidity, and nulliparous were associated risk factors with a higher incidence of anemia during pregnancy. Other risk factors comprised working, women in university, past history of anemia, obesity, women younger than 25 years, low income, longer menstrual cycle >5 days, bleeding during pregnancy, reduced birth spacing, a low level of education, and decreased intake of iron-rich foods. There should be ongoing health education and awareness campaigns on the value of using family planning and antenatal care services, as well as the provision of health facilities in remote areas to promote early booking and use of antenatal care services. Women should also have access to education and economic empowerment opportunities.
